# Crystal structure of octane-1,8-diaminium 4,4′-(diazene-1,2-di­yl)dibenzoate monohydrate

**DOI:** 10.1107/S2056989018006187

**Published:** 2018-04-27

**Authors:** Igor Elkin, Thierry Maris, Jan-Constantin Christopherson, Tristan H. Borchers, Christopher J. Barrett

**Affiliations:** a Chemistry Department, McGill University, Montreal, Quebec, H3A 0B8, Canada; b Department of Chemistry, Université de Montréal, Montreal, Quebec, H3C 3J7, Canada

**Keywords:** octane-1,8-diaminium 4,4′-azinodibenzoate, crystal structure, ionic pseudo-polymer, 4,4′-azinodi­benzoic acid

## Abstract

The title salt, C_8_H_22_N_2_^2+^·C_14_H_8_N_2_O_4_^2−^·H_2_O, represents a pseudo-polymer ionic material, resulting from the self-organizing behavior of 4,4′-azinodibenzoate dianions and doubly protonated, 1,8-diaminium-octane cations in aqueous solution. The asymmetric unit consists of two halves of octane 1,8-diaminium cations (the complete cations are both generated by crystallographic inversion symmetry), a 4,4′-azinodibenzoate anion [dihedral angle between the aromatic rings = 10.22 (4)°] and a water mol­ecule of crystallization. One of the cations is in a fully extended linear conformation while the second one has a terminal C—C—C—N *gauche* conformation. In the crystal, the cations, anions and water mol­ecules are linked into a three-dimensional network *via* a complex pattern of charge-assisted N—H⋯O and O—H⋯O hydrogen bonds.

## Abstract 2

The preparation and the crystal structure of octane-1,8-diaminium 4,4′-azinodibenzoate monohydrate are reported.

## Chemical context   

Ionic pseudo-polymers auto-assembled from oppositely charged organic mol­ecules are of emerging inter­est for many potential application fields (Webber *et al.*, 2016 ▸; Mann, 2009 ▸). It is reasonable to expect that the presence of azo­benzene moieties in such materials creates the necessary precondition for achieving their reversible photosensitivity (Bushuyev *et al.*, 2016 ▸, 2018 ▸). In this context, we report the synthesis and structure of octane-1,8-diaminium 4,4′-(diazene-1,2-di­yl)dibenzoate monohydrate, (I), formed by the crystallization of bianionic 4,4′-azinodi­benzoic acid and bicationic 1,8-di­amino­octane in aqueous solution.
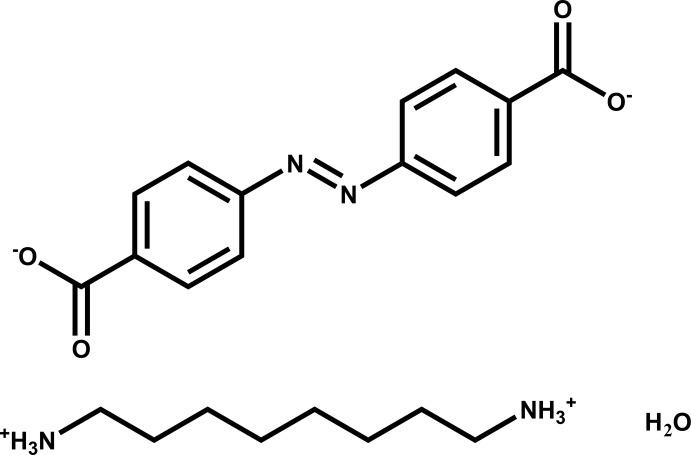


## Structural commentary   

**Figure 1:**
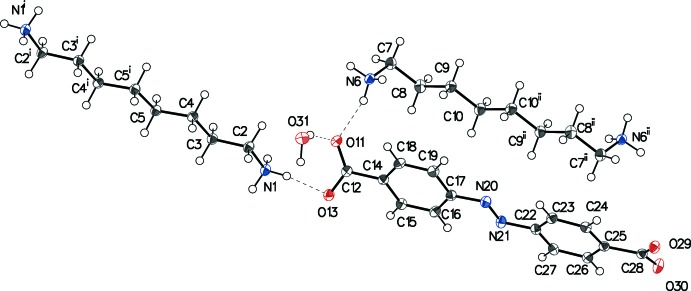
Mol­ecular structure and atom-labelling scheme of (I). Displacement ellipsoids are drawn at the 50% probability level and hydrogen bonds are shown as dotted lines. [Symmetry codes: (i) 1 − *x*, −*y*, 2 − *z*; (ii) −*x*, 1 − *y*, −*z*.]

The asymmetric unit (Fig. 1 ▸) consists of two halves of octane 1,8-diaminium dications, one 4,4′-azinodi­benzoic dianion and one water mol­ecule. Bond lengths and angles of the dication and dianion are in the expected ranges. One of the octane 1,8-diaminium dications shows a fully extended *all-trans* conformation with torsion angles close to 180° (Table 1 ▸). The second cation has its two terminal torsion angles N6—C7—C8—C9 synclinal with a value of −76.89 (12)°. The fully extended conformation corresponds to the most stable one, compared to the arrangement with synclinal torsion angles, as shown from DFT calculations and a database survey performed on α,ω-alkyl­diaminium cations (Brozdowska & Chojnacki, 2017 ▸). The less energetically favorable *gauche* conformation is presumably stabilized by the charge-assisted hydrogen-bonded network.

The geometry of the 4,4′-azinodi­benzoic dianion is characterized by the presence of two benzoic acid residues linked *via* a *trans*-configurated azo group is consistent with known data (Fernández *et al.*, 2015 ▸; Sahoo *et al.*, 2012 ▸). The angle between the phenyl rings of 10.22 (4)° is consistent with a small deviation from planarity of the azo­benzene moiety. The carboxyl­ate groups are inclined to the mol­ecular mean plane at angles of 38.40 (3)° (O11/C12/O13) and 16.53 (5)° (O29/C28/O30).

## Supra­molecular features   

**Figure 2:**
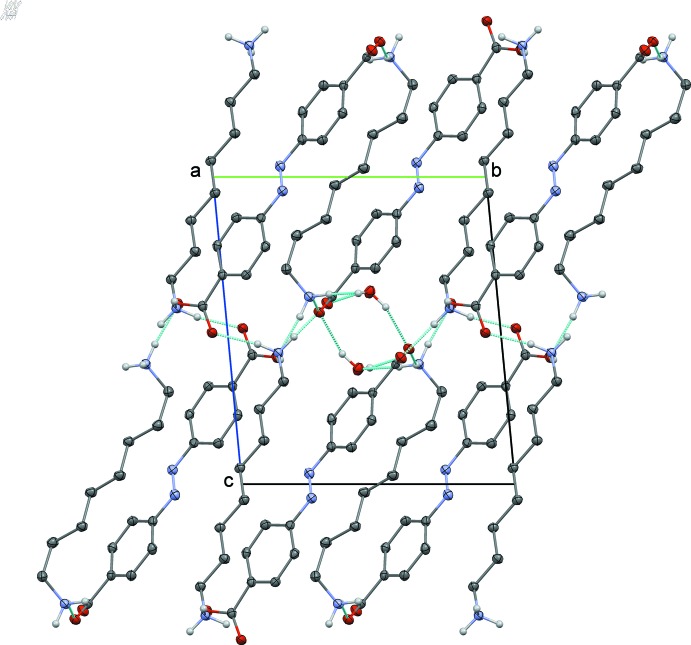
Projection along the *b*-axis direction showing the packing in layers consolidated by the hydrogen-bond network (dotted lines). Hydrogen atoms not involved in hydrogen bonds and hanging hydrogen bonds are omitted for clarity.

**Figure 3:**
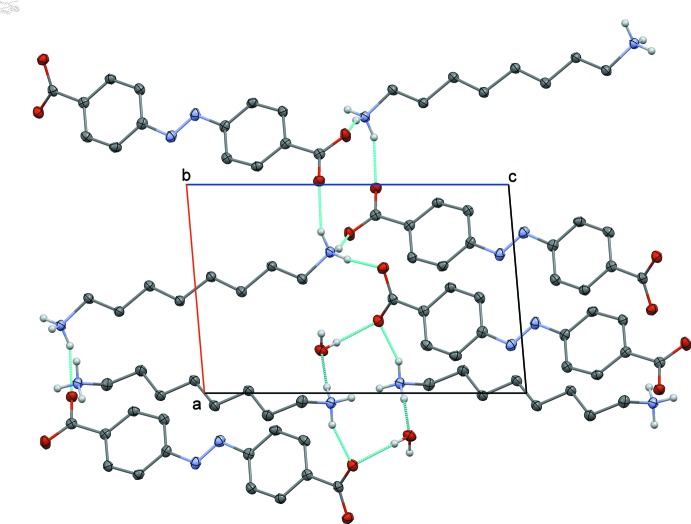
Partial packing view along the *b*-axis direction showing the 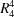
(12) graph-set motifs. Hydrogen atoms not involved in hydrogen bonds and hanging hydrogen bonds have been omitted for clarity.

In addition to the electrostatic inter­actions, the anions and cations are connected by charge-assisted N—H⋯O hydrogen bonds (Table 2 ▸). The complex pattern of hydrogen bonds also includes the water mol­ecules. Therefore, the 4,4′-azinodi­benzoic dianion is linked through hydrogen bonds with three cations on one side and with two cations and two water mol­ecules on the other side. Anions and cation stack in two-dimensional arrays in the *ab* plane separated by a zone with the hydrogen-bonded network involving the ionized amino and carb­oxy­lic groups and the water mol­ecules (Fig. 2 ▸). This network contains two 12-membered rings comprising either two cations and two anions or two cations, two anions and two water mol­ecules (Fig. 3 ▸), according to the graph set descriptor 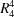
(12) (Etter *et al.*, 1990 ▸).

## Database survey   

A search in the Cambridge Structural Database (Version 5.39 with one update; Groom *et al.*, 2016 ▸) returned 48 entries for octane-1,8-diaminium compounds. These include simple halide salts (Brisson & Brisse, 1984 ▸; van Blerk & Kruger, 2007 ▸; van Megen & Reiss, 2013 ▸); metal halide salts (Kessentini *et al.*, 2011 ▸) comprising lead halide complexes (Lemmerer & Billing, 2012 ▸; Smith *et al.*, 2017 ▸), and more complex systems where the diaminium cations are encapsulated in a macrocycle (Kim *et al.*, 2009 ▸; Yu *et al.*, 2014 ▸). A similar search for 4,4′-azinodi­benzoic acid and its salts returned 43 entries, including the structure of the simple acid (Yu & Liu, 2009 ▸). The dianion has been also used as linker to prepare MOF or coordination frameworks (see, for example, Hou *et al.*, 2013 ▸, Zhang *et al.*, 2016 ▸, Guo *et al.*, 2013 ▸ and Deng *et al.*, 2015 ▸), and co-crystallized to give gelator salts (Sahoo & Dastidar, 2012 ▸; Sahoo *et al.*, 2012 ▸) or supra­molecular assemblies (Beatty *et al.*, 2002 ▸; Yu *et al.*, 2011 ▸).

## Synthesis and crystallization   

Crystals of the title compound were obtained by the dropwise addition with intensive stirring of 5 ml of 0.10 *M* aqueous 1,8-octa­methyl­enedi­amine into 25 ml of 0.02 *M* aqueous 4,4′-di­carb­oxy­azo­benzene disodium salt at room temperature. The final solution (pH 12.5) was allowed to partly evaporate at room temperature and atmospheric pressure. The resulting orange oblong crystals in the form of thin narrow leaves up to 1 cm long were gently removed from the liquid phase and air-dried on filter paper.

## Refinement   

Crystal data, data collection and structure refinement details are summarized in Table 3 ▸. Hydrogen atoms bound to nitro­gen or oxygen atoms were located from difference syntheses and refined without any restraints. Hydrogen atoms linked to carbon atoms were included using an appropriate riding model (AFIX 43 and AFIX 23 for aromatic and methyl­ene hydrogen atoms respectively) with C—H = 0.95–0.99 Å and *U*_iso_(H) = 1.2*U*_eq_(C).

## Supplementary Material

Crystal structure: contains datablock(s) I. DOI: 10.1107/S2056989018006187/hb7747sup1.cif

Structure factors: contains datablock(s) I. DOI: 10.1107/S2056989018006187/hb7747Isup2.hkl

Supporting information file. DOI: 10.1107/S2056989018006187/hb7747Isup3.cml

CCDC reference: 1839024

Additional supporting information:  crystallographic information; 3D view; checkCIF report

## Figures and Tables

**Table 1:** Selected torsion angles (°)

C2—C3—C4—C5	−177.97 (9)	C7—C8—C9—C10	−179.03 (9)
C3—C4—C5—C5^i^	178.25 (10)	C8—C9—C10—C10^ii^	178.30 (11)
N1—C2—C3—C4	177.78 (9)	N6—C7—C8—C9	−76.89 (12)

Symmetry codes: (i) 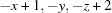; (ii) 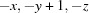.

**Table 2:** Hydrogen-bond geometry (Å, °)

*D*—H⋯*A*	*D*—H	H⋯*A*	*D*⋯*A*	*D*—H⋯*A*
N1—H1*A*⋯O30^iii^	0.895 (17)	1.897 (17)	2.7796 (13)	168.3 (14)
N1—H1*B*⋯O13	0.916 (16)	1.881 (17)	2.7942 (12)	175.0 (14)
N1—H1*C*⋯O29^iv^	0.918 (16)	1.970 (16)	2.8579 (12)	162.5 (13)
N6—H6*A*⋯O31^v^	0.921 (16)	1.912 (16)	2.8296 (13)	174.0 (13)
N6—H6*B*⋯O11	0.921 (16)	1.879 (16)	2.7735 (12)	163.2 (13)
N6—H6*C*⋯O29^vi^	0.947 (16)	1.849 (17)	2.7847 (12)	169.0 (14)
N6—H6*C*⋯O30^vi^	0.947 (16)	2.590 (16)	3.0880 (12)	113.2 (11)
O31—H31*A*⋯O13^vii^	0.89 (2)	1.88 (2)	2.7418 (11)	165.2 (17)
O31—H31*B*⋯O11	0.88 (2)	1.97 (2)	2.8184 (11)	162.2 (16)

Symmetry codes: (iii) 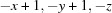; (iv) 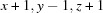; (v) 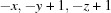; (vi) 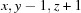; (vii) 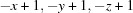.

**Table 3:** Experimental details

Crystal data	
Chemical formula	C_8_H_22_N_2_^2+^·C_14_H_8_N_2_O_4_^2−^·H_2_O
*M*_r_	432.51
Crystal system, space group	Triclinic, *P* 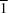
Temperature (K)	100
*a*, *b*, *c* (Å)	8.3604 (1), 11.4100 (2), 12.4661 (2)
α, β, γ (°)	83.001 (1), 83.364 (1), 73.973 (1)
*V* (Å^3^)	1130.24 (3)
*Z*	2
Radiation type	Cu *K*α
μ (mm^−1^)	0.75
Crystal size (mm)	0.4 × 0.4 × 0.1

Data collection	
Diffractometer	Bruker APEXII CCD
Absorption correction	Multi-scan (*SADABS*; Krause *et al.*, 2015 ▸)
*T*_min_, *T*_max_	0.525, 0.753
No. of measured, independent and observed [*I* > 2σ(*I*)] reflections	28413, 4048, 3920
*R*_int_	0.046
(sin θ/λ)_max_ (Å^−1^)	0.602

Refinement	
*R*[*F* ^2^ > 2σ(*F* ^2^)], *wR*(*F* ^2^), *S*	0.037, 0.096, 1.05
No. of reflections	4048
No. of parameters	313
H-atom treatment	H atoms treated by a mixture of independent and constrained refinement
Δρ_max_, Δρ_min_ (e Å^−3^)	0.20, −0.30

Computer programs: *APEX2* and *SAINT* (Bruker, 2013 ▸), *SHELXT* (Sheldrick, 2015*a* ▸), *SHELXL2018* (Sheldrick, 2015*b* ▸), *OLEX2* (Dolomanov *et al.*, 2009 ▸), *Mercury* (Macrae *et al.*, 2008 ▸) and *publCIF* (Westrip, 2010 ▸).

## supplementary crystallographic information

### Crystal data

**Table:**

C_8_H_22_N_2_^2+^·C_14_H_8_N_2_O_4_^2^^−^·H_2_O	*Z* = 2
*M**_r_* = 432.51	*F*(000) = 464
Triclinic, *P*1	*D*_x_ = 1.271 Mg m^−^^3^
*a* = 8.3604 (1) Å	Cu *K*α radiation, λ = 1.54184 Å
*b* = 11.4100 (2) Å	Cell parameters from 3920 reflections
*c* = 12.4661 (2) Å	θ = 4.1–68.1°
α = 83.001 (1)°	µ = 0.75 mm^−^^1^
β = 83.364 (1)°	*T* = 100 K
γ = 73.973 (1)°	Block, orange
*V* = 1130.24 (3) Å^3^	0.4 × 0.4 × 0.1 mm

### Data collection

**Table:**

Bruker APEXII CCD diffractometer	4048 independent reflections
Radiation source: fine-focus sealed tube	3920 reflections with *I* > 2σ(*I*)
Graphite monochromator	*R*_int_ = 0.046
φ and ω scans	θ_max_ = 68.1°, θ_min_ = 4.1°
Absorption correction: multi-scan (SADABS; Krause *et al.*, 2015)	*h* = −10→10
*T*_min_ = 0.525, *T*_max_ = 0.753	*k* = −13→13
28413 measured reflections	*l* = −14→14

### Refinement

**Table:**

Refinement on *F*^2^	Hydrogen site location: mixed
Least-squares matrix: full	H atoms treated by a mixture of independent and constrained refinement
*R*[*F*^2^ > 2σ(*F*^2^)] = 0.037	*w* = 1/[σ^2^(*F*_o_^2^) + (0.0541*P*)^2^ + 0.3252*P*] where *P* = (*F*_o_^2^ + 2*F*_c_^2^)/3
*wR*(*F*^2^) = 0.096	(Δ/σ)_max_ = 0.001
*S* = 1.05	Δρ_max_ = 0.20 e Å^−^^3^
4048 reflections	Δρ_min_ = −0.30 e Å^−^^3^
313 parameters	Extinction correction: SHELXL2018 (Sheldrick, 2015b), Fc^*^=kFc[1+0.001xFc^2^λ^3^/sin(2θ)]^-1/4^
0 restraints	Extinction coefficient: 0.0427 (17)
Primary atom site location: structure-invariant direct methods	

### Special details

**Table:**

Geometry. All esds (except the esd in the dihedral angle between two l.s. planes) are estimated using the full covariance matrix. The cell esds are taken into account individually in the estimation of esds in distances, angles and torsion angles; correlations between esds in cell parameters are only used when they are defined by crystal symmetry. An approximate (isotropic) treatment of cell esds is used for estimating esds involving l.s. planes.

### Fractional atomic coordinates and isotropic or equivalent isotropic displacement parameters (Å^2^)

**Table:**

	*x*	*y*	*z*	*U*_iso_*/*U*_eq_	
C12	0.44977 (13)	0.39551 (9)	0.39040 (8)	0.0164 (2)	
C14	0.39242 (13)	0.48992 (10)	0.29711 (9)	0.0163 (2)	
C15	0.50609 (13)	0.50353 (10)	0.20773 (9)	0.0180 (2)	
H15	0.618450	0.454880	0.207597	0.022*	
C16	0.45684 (14)	0.58671 (10)	0.11990 (9)	0.0187 (2)	
H16	0.534114	0.593956	0.058933	0.022*	
C17	0.29240 (14)	0.66026 (10)	0.12125 (9)	0.0175 (2)	
C18	0.17852 (14)	0.64902 (10)	0.21041 (9)	0.0193 (2)	
H18	0.067342	0.699946	0.211663	0.023*	
C19	0.22842 (14)	0.56296 (10)	0.29746 (9)	0.0185 (2)	
H19	0.150301	0.553905	0.357480	0.022*	
C22	0.27923 (14)	0.83834 (10)	−0.13411 (9)	0.0169 (2)	
C23	0.12601 (14)	0.92810 (10)	−0.13255 (9)	0.0182 (2)	
H23	0.049801	0.933790	−0.069462	0.022*	
C24	0.08731 (13)	1.00833 (10)	−0.22421 (9)	0.0174 (2)	
H24	−0.015163	1.070596	−0.222999	0.021*	
C25	0.19653 (13)	0.99937 (9)	−0.31875 (9)	0.0162 (2)	
C26	0.35000 (14)	0.91128 (10)	−0.31874 (9)	0.0173 (2)	
H26	0.426383	0.905667	−0.381738	0.021*	
C27	0.39101 (13)	0.83176 (10)	−0.22644 (9)	0.0178 (2)	
H27	0.496160	0.772422	−0.226335	0.021*	
C28	0.14659 (13)	1.08221 (10)	−0.42094 (9)	0.0168 (2)	
N20	0.23031 (12)	0.75035 (8)	0.03481 (8)	0.0199 (2)	
N21	0.33560 (12)	0.75025 (8)	−0.04586 (7)	0.0194 (2)	
O11	0.34625 (9)	0.34229 (7)	0.44062 (6)	0.0200 (2)	
O13	0.59935 (9)	0.37427 (7)	0.41176 (6)	0.0209 (2)	
O29	0.01715 (9)	1.17097 (7)	−0.41274 (6)	0.0204 (2)	
O30	0.23647 (10)	1.05688 (7)	−0.50725 (6)	0.0234 (2)	
C2	0.54646 (13)	0.18776 (10)	0.66601 (9)	0.0181 (2)	
H2A	0.442430	0.177349	0.642576	0.022*	
H2B	0.521051	0.269756	0.692620	0.022*	
C3	0.60840 (13)	0.08987 (10)	0.75674 (9)	0.0183 (2)	
H3A	0.715478	0.098563	0.776743	0.022*	
H3B	0.630505	0.008358	0.729672	0.022*	
C4	0.48635 (13)	0.09539 (10)	0.85797 (8)	0.0176 (2)	
H4A	0.380562	0.083043	0.839346	0.021*	
H4B	0.461117	0.177451	0.884428	0.021*	
C5	0.55783 (13)	−0.00224 (10)	0.94788 (8)	0.0178 (2)	
H5A	0.580205	−0.084114	0.921493	0.021*	
H5B	0.665684	0.008692	0.964131	0.021*	
N1	0.67578 (12)	0.17874 (9)	0.57222 (8)	0.0177 (2)	
H1A	0.6930 (19)	0.1068 (16)	0.5450 (12)	0.033 (4)*	
H1B	0.6457 (19)	0.2412 (15)	0.5188 (12)	0.031 (4)*	
H1C	0.777 (2)	0.1830 (13)	0.5905 (12)	0.028 (4)*	
C7	0.04089 (14)	0.23686 (10)	0.30051 (9)	0.0206 (3)	
H7A	−0.076237	0.235667	0.296577	0.025*	
H7B	0.107206	0.151394	0.317135	0.025*	
C8	0.10729 (14)	0.28667 (10)	0.19086 (9)	0.0222 (3)	
H8A	0.134670	0.221260	0.141092	0.027*	
H8B	0.212435	0.306963	0.199818	0.027*	
C9	−0.01161 (13)	0.39982 (10)	0.13799 (9)	0.0194 (2)	
H9A	−0.117543	0.380517	0.129346	0.023*	
H9B	−0.037363	0.466406	0.186456	0.023*	
C10	0.06004 (14)	0.44447 (10)	0.02760 (9)	0.0210 (3)	
H10A	0.089089	0.376931	−0.020020	0.025*	
H10B	0.164268	0.465977	0.036703	0.025*	
N6	0.04744 (12)	0.31029 (9)	0.39017 (7)	0.0178 (2)	
H6A	−0.0289 (18)	0.3860 (14)	0.3860 (11)	0.026 (3)*	
H6B	0.153 (2)	0.3200 (13)	0.3926 (12)	0.029 (4)*	
H6C	0.0223 (19)	0.2670 (14)	0.4569 (13)	0.033 (4)*	
O31	0.20434 (10)	0.46567 (8)	0.62560 (7)	0.0235 (2)	
H31A	0.281 (2)	0.5069 (17)	0.6193 (14)	0.049 (5)*	
H31B	0.241 (2)	0.4140 (17)	0.5755 (15)	0.048 (5)*	

### Atomic displacement parameters (Å^2^)

**Table:**

	*U*^11^	*U*^22^	*U*^33^	*U*^12^	*U*^13^	*U*^23^
C12	0.0197 (6)	0.0127 (5)	0.0160 (5)	−0.0030 (4)	0.0010 (4)	−0.0038 (4)
C14	0.0187 (5)	0.0134 (5)	0.0176 (5)	−0.0050 (4)	−0.0011 (4)	−0.0029 (4)
C15	0.0165 (5)	0.0163 (5)	0.0192 (5)	−0.0014 (4)	−0.0007 (4)	−0.0022 (4)
C16	0.0201 (6)	0.0182 (5)	0.0170 (5)	−0.0046 (4)	0.0020 (4)	−0.0024 (4)
C17	0.0209 (6)	0.0135 (5)	0.0184 (5)	−0.0048 (4)	−0.0030 (4)	−0.0012 (4)
C18	0.0160 (5)	0.0161 (5)	0.0234 (6)	−0.0009 (4)	−0.0009 (4)	−0.0015 (4)
C19	0.0188 (5)	0.0161 (5)	0.0197 (5)	−0.0048 (4)	0.0029 (4)	−0.0017 (4)
C22	0.0203 (5)	0.0137 (5)	0.0174 (5)	−0.0041 (4)	−0.0041 (4)	−0.0023 (4)
C23	0.0193 (5)	0.0183 (5)	0.0168 (5)	−0.0053 (4)	0.0003 (4)	−0.0027 (4)
C24	0.0156 (5)	0.0147 (5)	0.0210 (5)	−0.0017 (4)	−0.0020 (4)	−0.0026 (4)
C25	0.0177 (5)	0.0136 (5)	0.0183 (5)	−0.0051 (4)	−0.0028 (4)	−0.0021 (4)
C26	0.0186 (5)	0.0171 (5)	0.0164 (5)	−0.0044 (4)	−0.0005 (4)	−0.0034 (4)
C27	0.0170 (5)	0.0155 (5)	0.0198 (5)	−0.0006 (4)	−0.0036 (4)	−0.0036 (4)
C28	0.0180 (5)	0.0147 (5)	0.0186 (5)	−0.0057 (4)	−0.0017 (4)	−0.0014 (4)
N20	0.0213 (5)	0.0176 (5)	0.0197 (5)	−0.0042 (4)	−0.0027 (4)	0.0006 (4)
N21	0.0234 (5)	0.0167 (5)	0.0170 (5)	−0.0031 (4)	−0.0027 (4)	−0.0015 (4)
O11	0.0222 (4)	0.0189 (4)	0.0190 (4)	−0.0074 (3)	−0.0003 (3)	0.0013 (3)
O13	0.0196 (4)	0.0197 (4)	0.0220 (4)	−0.0042 (3)	−0.0038 (3)	0.0033 (3)
O29	0.0190 (4)	0.0174 (4)	0.0211 (4)	−0.0002 (3)	−0.0014 (3)	0.0014 (3)
O30	0.0284 (4)	0.0192 (4)	0.0169 (4)	0.0010 (3)	0.0015 (3)	0.0007 (3)
C2	0.0176 (5)	0.0178 (5)	0.0170 (5)	−0.0032 (4)	0.0020 (4)	−0.0009 (4)
C3	0.0162 (5)	0.0195 (5)	0.0176 (5)	−0.0035 (4)	0.0001 (4)	−0.0003 (4)
C4	0.0174 (5)	0.0183 (5)	0.0168 (5)	−0.0049 (4)	0.0007 (4)	−0.0018 (4)
C5	0.0178 (5)	0.0186 (6)	0.0165 (6)	−0.0044 (4)	0.0001 (5)	−0.0020 (4)
N1	0.0176 (5)	0.0169 (5)	0.0163 (5)	−0.0023 (4)	0.0001 (4)	0.0008 (4)
C7	0.0246 (6)	0.0164 (5)	0.0200 (6)	−0.0033 (4)	−0.0032 (4)	−0.0017 (4)
C8	0.0199 (6)	0.0231 (6)	0.0200 (6)	0.0002 (4)	−0.0004 (4)	−0.0035 (4)
C9	0.0178 (5)	0.0213 (6)	0.0170 (5)	−0.0026 (4)	0.0007 (4)	−0.0023 (4)
C10	0.0193 (5)	0.0241 (6)	0.0177 (6)	−0.0037 (5)	0.0023 (4)	−0.0025 (5)
N6	0.0184 (5)	0.0160 (5)	0.0179 (5)	−0.0040 (4)	−0.0016 (4)	0.0008 (4)
O31	0.0213 (4)	0.0217 (4)	0.0262 (4)	−0.0041 (3)	0.0024 (3)	−0.0056 (3)

### Geometric parameters (Å, º)

**Table:**

C12—C14	1.5103 (14)	C2—N1	1.4907 (13)
C12—O11	1.2578 (13)	C3—H3A	0.9900
C12—O13	1.2599 (14)	C3—H3B	0.9900
C14—C15	1.4011 (15)	C3—C4	1.5244 (14)
C14—C19	1.3939 (15)	C4—H4A	0.9900
C15—H15	0.9500	C4—H4B	0.9900
C15—C16	1.3800 (15)	C4—C5	1.5289 (15)
C16—H16	0.9500	C5—C5^i^	1.525 (2)
C16—C17	1.3984 (16)	C5—H5A	0.9900
C17—C18	1.3951 (15)	C5—H5B	0.9900
C17—N20	1.4325 (14)	N1—H1A	0.895 (17)
C18—H18	0.9500	N1—H1B	0.916 (16)
C18—C19	1.3909 (16)	N1—H1C	0.918 (16)
C19—H19	0.9500	C7—H7A	0.9900
C22—C23	1.4018 (16)	C7—H7B	0.9900
C22—C27	1.3920 (15)	C7—C8	1.5196 (16)
C22—N21	1.4253 (14)	C7—N6	1.4905 (14)
C23—H23	0.9500	C8—H8A	0.9900
C23—C24	1.3839 (16)	C8—H8B	0.9900
C24—H24	0.9500	C8—C9	1.5247 (15)
C24—C25	1.4008 (15)	C9—H9A	0.9900
C25—C26	1.3944 (16)	C9—H9B	0.9900
C25—C28	1.5172 (15)	C9—C10	1.5228 (15)
C26—H26	0.9500	C10—C10^ii^	1.527 (2)
C26—C27	1.3891 (15)	C10—H10A	0.9900
C27—H27	0.9500	C10—H10B	0.9900
C28—O29	1.2656 (13)	N6—H6A	0.921 (16)
C28—O30	1.2549 (13)	N6—H6B	0.921 (16)
N20—N21	1.2575 (14)	N6—H6C	0.947 (16)
C2—H2A	0.9900	O31—H31A	0.89 (2)
C2—H2B	0.9900	O31—H31B	0.88 (2)
C2—C3	1.5176 (15)		
			
O11—C12—C14	118.09 (9)	H3A—C3—H3B	107.7
O11—C12—O13	124.36 (10)	C4—C3—H3A	108.8
O13—C12—C14	117.54 (9)	C4—C3—H3B	108.8
C15—C14—C12	119.29 (9)	C3—C4—H4A	109.3
C19—C14—C12	121.45 (9)	C3—C4—H4B	109.3
C19—C14—C15	119.26 (10)	C3—C4—C5	111.41 (9)
C14—C15—H15	119.6	H4A—C4—H4B	108.0
C16—C15—C14	120.82 (10)	C5—C4—H4A	109.3
C16—C15—H15	119.6	C5—C4—H4B	109.3
C15—C16—H16	120.2	C4—C5—H5A	108.9
C15—C16—C17	119.54 (10)	C4—C5—H5B	108.9
C17—C16—H16	120.2	C5^i^—C5—C4	113.31 (11)
C16—C17—N20	123.64 (10)	C5^i^—C5—H5A	108.9
C18—C17—C16	120.26 (10)	C5^i^—C5—H5B	108.9
C18—C17—N20	116.10 (9)	H5A—C5—H5B	107.7
C17—C18—H18	120.1	C2—N1—H1A	109.6 (10)
C19—C18—C17	119.74 (10)	C2—N1—H1B	112.0 (9)
C19—C18—H18	120.1	C2—N1—H1C	113.2 (9)
C14—C19—H19	119.8	H1A—N1—H1B	109.5 (13)
C18—C19—C14	120.36 (10)	H1A—N1—H1C	107.0 (13)
C18—C19—H19	119.8	H1B—N1—H1C	105.4 (13)
C23—C22—N21	125.14 (10)	H7A—C7—H7B	107.8
C27—C22—C23	120.04 (10)	C8—C7—H7A	109.0
C27—C22—N21	114.81 (9)	C8—C7—H7B	109.0
C22—C23—H23	120.5	N6—C7—H7A	109.0
C24—C23—C22	119.07 (10)	N6—C7—H7B	109.0
C24—C23—H23	120.5	N6—C7—C8	112.95 (9)
C23—C24—H24	119.4	C7—C8—H8A	108.5
C23—C24—C25	121.19 (10)	C7—C8—H8B	108.5
C25—C24—H24	119.4	C7—C8—C9	114.99 (9)
C24—C25—C28	120.42 (9)	H8A—C8—H8B	107.5
C26—C25—C24	119.24 (10)	C9—C8—H8A	108.5
C26—C25—C28	120.32 (10)	C9—C8—H8B	108.5
C25—C26—H26	120.1	C8—C9—H9A	109.0
C27—C26—C25	119.89 (10)	C8—C9—H9B	109.0
C27—C26—H26	120.1	H9A—C9—H9B	107.8
C22—C27—H27	119.7	C10—C9—C8	112.75 (9)
C26—C27—C22	120.51 (10)	C10—C9—H9A	109.0
C26—C27—H27	119.7	C10—C9—H9B	109.0
O29—C28—C25	117.57 (9)	C9—C10—C10^ii^	113.21 (11)
O30—C28—C25	117.37 (9)	C9—C10—H10A	108.9
O30—C28—O29	125.06 (10)	C9—C10—H10B	108.9
N21—N20—C17	112.88 (9)	C10^ii^—C10—H10A	108.9
N20—N21—C22	114.90 (9)	C10^ii^—C10—H10B	108.9
H2A—C2—H2B	108.1	H10A—C10—H10B	107.7
C3—C2—H2A	109.6	C7—N6—H6A	112.5 (9)
C3—C2—H2B	109.6	C7—N6—H6B	111.9 (9)
N1—C2—H2A	109.6	C7—N6—H6C	108.3 (9)
N1—C2—H2B	109.6	H6A—N6—H6B	109.4 (13)
N1—C2—C3	110.15 (9)	H6A—N6—H6C	107.9 (12)
C2—C3—H3A	108.8	H6B—N6—H6C	106.5 (13)
C2—C3—H3B	108.8	H31A—O31—H31B	102.9 (16)
C2—C3—C4	113.74 (9)		
			
C12—C14—C15—C16	178.35 (9)	C25—C26—C27—C22	0.71 (16)
C12—C14—C19—C18	−179.80 (9)	C26—C25—C28—O29	−170.63 (9)
C14—C15—C16—C17	1.46 (16)	C26—C25—C28—O30	9.78 (15)
C15—C14—C19—C18	−0.49 (16)	C27—C22—C23—C24	0.85 (16)
C15—C16—C17—C18	−0.48 (16)	C27—C22—N21—N20	−175.22 (9)
C15—C16—C17—N20	179.03 (9)	C28—C25—C26—C27	−176.74 (9)
C16—C17—C18—C19	−0.97 (16)	N20—C17—C18—C19	179.48 (9)
C16—C17—N20—N21	4.90 (15)	N21—C22—C23—C24	179.78 (9)
C17—C18—C19—C14	1.45 (16)	N21—C22—C27—C26	178.99 (9)
C17—N20—N21—C22	179.88 (8)	O11—C12—C14—C15	−146.46 (10)
C18—C17—N20—N21	−175.57 (9)	O11—C12—C14—C19	32.86 (14)
C19—C14—C15—C16	−0.99 (16)	O13—C12—C14—C15	32.65 (14)
C22—C23—C24—C25	1.52 (16)	O13—C12—C14—C19	−148.03 (10)
C23—C22—C27—C26	−1.97 (16)	C2—C3—C4—C5	−177.97 (9)
C23—C22—N21—N20	5.80 (15)	C3—C4—C5—C5^i^	178.25 (10)
C23—C24—C25—C26	−2.76 (16)	N1—C2—C3—C4	177.78 (9)
C23—C24—C25—C28	175.60 (9)	C7—C8—C9—C10	−179.03 (9)
C24—C25—C26—C27	1.62 (15)	C8—C9—C10—C10^ii^	178.30 (11)
C24—C25—C28—O29	11.03 (14)	N6—C7—C8—C9	−76.89 (12)
C24—C25—C28—O30	−168.56 (10)		

Symmetry codes: (i) −*x*+1, −*y*, −*z*+2; (ii) −*x*, −*y*+1, −*z*.

### Hydrogen-bond geometry (Å, º)

**Table:**

*D*—H···*A*	*D*—H	H···*A*	*D*···*A*	*D*—H···*A*
N1—H1*A*···O30^iii^	0.895 (17)	1.897 (17)	2.7796 (13)	168.3 (14)
N1—H1*B*···O13	0.916 (16)	1.881 (17)	2.7942 (12)	175.0 (14)
N1—H1*C*···O29^iv^	0.918 (16)	1.970 (16)	2.8579 (12)	162.5 (13)
N6—H6*A*···O31^v^	0.921 (16)	1.912 (16)	2.8296 (13)	174.0 (13)
N6—H6*B*···O11	0.921 (16)	1.879 (16)	2.7735 (12)	163.2 (13)
N6—H6*C*···O29^vi^	0.947 (16)	1.849 (17)	2.7847 (12)	169.0 (14)
N6—H6*C*···O30^vi^	0.947 (16)	2.590 (16)	3.0880 (12)	113.2 (11)
O31—H31*A*···O13^vii^	0.89 (2)	1.88 (2)	2.7418 (11)	165.2 (17)
O31—H31*B*···O11	0.88 (2)	1.97 (2)	2.8184 (11)	162.2 (16)

Symmetry codes: (iii) −*x*+1, −*y*+1, −*z*; (iv) *x*+1, *y*−1, *z*+1; (v) −*x*, −*y*+1, −*z*+1; (vi) *x*, *y*−1, *z*+1; (vii) −*x*+1, −*y*+1, −*z*+1.
